# Structure Determination in Cell Slices using 2D Template Matching

**DOI:** 10.1063/4.0000822

**Published:** 2025-10-27

**Authors:** Johannes Elferich, Stephen Diggs, Lingli Kong, Emily Plumb, Robert Arkowitz, Nikolaus Grigorieff

**Affiliations:** 1 UMass Chan Medical School, Worcester, MA, USA; 2 Université Côte d'Azur, Nice, Alpes-Maritimes, France

## Abstract

Cryogenic electron microscopy can be used to determine the structure of biomolecules in their native cellular environment, provided that sufficiently thin slices of cells can be prepared. However, compared to single-particle reconstruction, significant challenges remain, including lower throughput in data acquisition and difficulties in identifying targets within the dense cellular milieu.

An emerging approach to overcome these challenges is 2D template matching (2DTM), which employs a brute-force cross-correlation search between untilted exposures of cellular samples and all possible projections of a template to detect targets. From the resulting detections, high- resolution reconstructions can be generated using standard single-particle approaches. A key concern in this approach is template bias, which can dominate the reconstruction. In practice, regions of biological interest can be removed from the template to obtain bias-free reconstructions of these regions. Since data collection does not require tilting, we can routinely acquire more than 10,000 exposures per day. Another advantage of 2DTM is that it lends itself to montaged imaging approaches, thereby maximizing the use of cell slices, whose preparation is time consuming. In this talk, I will present our current best practices for 2DTM data collection and processing, along with recent work on benchmarking sample damage in cryogenic sections and characterizing translational states in the pathogenic fungus Candida albicans.

